# Elevated Glucose on Admission Was an Independent Risk Factor for 30-Day Major Adverse Cardiovascular Events in Patients with STEMI but Not NSTEMI

**DOI:** 10.31083/j.rcm2502046

**Published:** 2024-01-29

**Authors:** Linlin Ma, Yanan Li, Junyu Pei, Xiaopu Wang, Keyang Zheng, Zixu Zhao, Jiafu Yan, Rufei Liu, Tianzhu Zhao, Yuxuan Wei, Wenli Cheng

**Affiliations:** ^1^Center of Hypertension, Beijing Anzhen Hospital, Capital Medical University, 100029 Beijing, China; ^2^School of Public Health, Brown University, Providence, RI 02903, USA; ^3^Department of Cardiovascular Medicine, The Second Xiangya Hospital, Central South University, 410011 Changsha, Hunan, China; ^4^Department of Biochemistry and Molecular Biology, The Libin Cardiovascular Institute of Alberta, The University of Calgary, Health Sciences Center, Calgary, AB T2N 4N1, Canada; ^5^Department of Cardiovascular Medicine, Beijing Anzhen Hospital, Capital Medical University, 100029 Beijing, China; ^6^Department of Cardiology, Affiliated Hospital of Yangzhou University, Yangzhou University, 225000 Yangzhou, Jiangsu, China

**Keywords:** glucose, major adverse cardiovascular events, ST-elevation myocardial infarction, non-ST-elevation myocardial infarction

## Abstract

**Background:**

The purpose of this study was to evaluate the impact of glucose levels on admission, on the risk of 30-day major adverse cardiovascular events (MACEs) in patients with acute myocardial infarction (AMI), and to assess the difference in outcome between ST-segment elevation myocardial infarction (STEMI) and non-ST-segment elevation myocardial infarction (NSTEMI) patients.

**Methods:**

This study was a post hoc analysis of the Acute Coronary Syndrome Quality Improvement in Kerala Study, and 13,398 participants were included in the final analysis. Logistic regression models were used to assess the association between glucose levels on admission and the risk of 30-day MACEs, adjusting for potential confounders.

**Results:**

Participants were divided according to the glucose quintiles. There was a positive linear association between glucose levels at admission and the risk of 30-day MACEs in AMI patients [adjusted OR (95% CI): 1.05 (1.03, 1.07), *p* < 0.001]. Compared to participants with an admission glucose between 5.4 and 6.3 mmol/L, participants with the highest quintile of glucose level (≥10.7 mmol/L) were associated with increased risk of 30-day MACEs in the fully adjusted logistic regression model [adjusted OR (95% CI): 1.82 (1.33, 2.50), *p* < 0.001]. This trend was more significant in patients with STEMI (*p* for interaction = 0.036).

**Conclusions:**

In patients with AMI, elevated glucose on admission was associated with an increased risk of 30-day MACEs, but only in patients with STEMI.

## 1. Introduction

Cardiovascular disease (CVD) is the leading cause of mortality in India, mainly due to ischemic heart disease (IHD) [1, 2]. As a severe subtype of coronary heart disease, acute myocardial infarction (AMI) was more common in India than in other countries due to a combination of a large population and genetic background [3, 4].

Previous studies found that hypertension, diabetes, physical activity, and moderate alcohol use were independent risk factors for coronary heart disease (CHD) in both males and females [5]. Early identification of acute coronary syndrome (ACS) patients with poor prognosis is very important. Different subtypes of ACS, such as ST-segment elevation myocardial infarction (STEMI) and non-ST-segment elevation myocardial infarction (NSTEMI), have different pathophysiological and clinical features, and their prognosis is also different [6, 7]. Myocardial troponin peak (cTn), as one of the prognostic factors of AMI, had a different prognostic value for different types of AMI [8].

Abnormally elevated blood glucose was common in patients with AMI [9, 10]. Previous studies had suggested that elevated glucose levels on admission were associated with an unfavorable prognosis in patients with AMI [11, 12]. However, the role of admission glucose levels was not previously investigated in STEMI and NSTEMI patients specifically. Therefore, the purpose of this study was to evaluate the impact of glucose levels on admission on the risk of 30-day major adverse cardiovascular events (MACEs) in AMI patients, and to assess the difference in outcome between STEMI and NSTEMI patients.

## 2. Methods 

### 2.1 Data Source and Study Participants

The data analyzed in this study were from the Acute Coronary Syndrome Quality Improvement in Kerala (ACS-QUIK) Study, which was available on the National Heart, Lung and Blood Institute website with reasonable application (https://biolincc.nhlbi.nih.gov/studies/acs_quik/). The rationale and main result of the ACS-QUIK Study have been published previously [13, 14]. In brief, the ACS-QUIK Study was a cluster-randomized, stepped-wedged clinical trial conducted in 63 hospitals in Kerala, India, from November 10, 2014 to November 9, 2016. The aim of this study was to assess whether a locally adapted quality improvement tool kit could improve the process of care measures and clinical outcomes for patients with acute myocardial infarction. The ACS-QUIK Study was approved by the ethics committees of local, national and international agencies and approved by the Indian Health Ministry Screening Committee. All participants or their representatives provided written informed consent to participate in the trial. Among 21,374 patients with acute myocardial infarction enrolled in this trial, the locally adopted quality improvement kits did not reduce the incidence of 30-day MACEs compared with conventional care.

This analysis was to evaluate the effect of the glucose level on admission on the incidence of 30-day MACEs in patients with acute myocardial infarction at ACS-QUIK Study and to assess the difference in outcome between STEMI and NSTEMI patients. After 7976 participants without glucose on admission were excluded, we finally included 13,398 participants in this analysis and divided them according to the glucose quintiles [Q1 (≤5.3), Q2 (5.4–6.3), Q3 (6.4–7.8), Q4 (7.9–10.6), Q5 (≥10.7), with Q2 (5.4–6.3) as reference]. The flowchart of analysis of this study is shown in **Supplementary Fig. 1**.

### 2.2 Baseline Parameters and Study Outcome

Other baseline parameters included demographic data (age, gender, weight, smoking or tobacco), examination at admission (systolic blood pressure (SBP), heart rate, high density lipoprotein cholesterol (HDL-C), low density lipoprotein cholesterol (LDL-C), triglycerides), left ventricular ejection fraction (LVEF) and LVEF category, prior comorbidities [hypertension, peripheral artery disease (PAD), diabetes, prior transient ischemic attack (TIA) or stroke], type of myocardial infarction (NSTEMI, STEMI), Killip class at admission and cardiac status, medication at admission (Beta Blocker, antiplatelet), symptom onset to arrival time, and percutaneous coronary intervention (PCI).

The study outcome was 30-day MACEs, including morality, stroke and reinfarction. The diagnostic criteria for reinfarction were defined according to the Third Universal Definition of Myocardial Infarction [15].

### 2.3 Statistical Analyses

The categorial variables were described statistically by frequency and percentage, and the continuous variables were described statistically by mean ± standard deviation (for normal distribution) or median (P25, P75) (for skewness distribution), respectively. Analysis of variance (ANOVA) or nonparametric test was used for testing inter-group differences of continuous variables, and chi-square test or Fisher test was used for categorical variables. The association of 30-day MACEs with the baseline glucose quintiles was assessed by three logistic regression analysis models. All covariates that might influence short-term outcomes in patients with acute MI were included in the analysis. The diagnosis of multicollinearity in the covariates included in the logistic model were measured by the variance inflation factor (VIF) (**Supplementary Table 1**). If VIF ≥5, multicollinearity existed among the covariates and the corresponding variables were eliminated. Covariates with a *p* value of the regression coefficient less than 0.1 were adjusted in the full model (**Supplementary Table 2**). According to the STROBE statement [16], model 1 was unadjusted model, model 2 was minimally adjusted for intervention, age, sex and SBP, and model 3 was fully adjusted for intervention, age, sex, SBP, weight, heart rate, HDL-C, LDL-C, triglyceride (TG), smoking or tobacco, diabetes, hypertension, PAD, MI Type, time from symptom onset to arrival, prior TIA or stroke, cardiac arrest at admission, LVEF category, PCI, antiplatelet, beta blocker. The same analysis procedures were used to assess the relationship between MI type and 30-day MACEs, adjusting for all the covariates mentioned above, including glucose in model 3. We also used a generalized additive model (GAM) to visualize the dose-response relationship between glucose levels at admission and the risk of 30-day MACEs and stratified the dose-response relationship by MI type (covariates in model 3 were adjusted). We also analyzed the interaction between glucose levels at admission and prespecified subgroups on the risk of 30-day MACEs. All analyses were performed using the statistical software package R version 4.0.0 (The R Foundation; http://www.R-project.org). Statistical significance was set at *p* < 0.05.

## 3. Results

### 3.1 Baseline Characteristics and Crude Outcomes

Table 1 shows the baseline characteristics and crude outcomes across the glucose quintiles. As expected, the higher glucose quartile was associated with a higher incidence of 30-day MACE and a higher prevalence of diabetes. Participants in the higher glucose quartile had a higher proportion of females, a higher SBP and heart rate at admission, higher Killip class (II~IV), a lower rate of smoking or tobacco and a higher prevalence of hypertension, PAD and stroke, as well as a lower LVEF and a higher incidence of cardiac arrest than participants in the lower glucose quartile. There were significant inter-group differences in weight, HDL-C, triglyceride MI type and PCI therapy, while there were no inter-group differences in medication (beta blocker, antiplatelet) at admission and time from symptom onset to arrival.

**Table 1.** **Baseline characteristics and crude outcome according to glucose quintiles**.

Variables		Glucose at admission (mmol/L)					*p*-value
		Q1 (≤5.3)	Q2 (5.4–6.3)	Q3 (6.4–7.8)	Q4 (7.9–10.6)	Q5 (≥10.7)	
N		2644	2519	2856	2648	2731	
Intervention		1290 (51.21%)	1109 (41.94%)	1514 (53.01%)	1391 (52.53%)	1436 (52.58%)	<0.001
Age, y, mean ± SD		59.63 ± 12.87	59.76 ± 12.42	60.87 ± 12.13	60.75 ± 11.55	60.48 ± 11.28	<0.001
Age group, n (%)							0.160
	<65	1718 (64.98%)	1635 (64.91%)	1785 (62.50%)	1669 (63.03%)	1769 (64.77%)	
	≥65	926 (35.02%)	884 (35.09%)	1071 (37.50%)	979 (36.97%)	962 (35.23%)	
Sex, n (%)							<0.001
	Female	566 (21.41%)	532 (21.12%)	717 (25.11%)	685 (25.87%)	786 (28.78%)	
	Male	2078 (78.59%)	1987 (78.88%)	2139 (74.89%)	1963 (74.13%)	1945 (71.22%)	
SBP, mmHg, mean ± SD		137.29 ± 27.41	138.33 ± 26.97	140.31 ± 29.04	141.47 ± 30.68	142.06 ± 31.01	<0.001
Heart Rate, bpm, mean ± SD		77.72 ± 18.10	78.15 ± 17.73	79.79 ± 19.04	81.84 ± 20.10	85.10 ± 20.80	<0.001
Weight, kg, mean ± SD		62.75 ± 9.03	63.61 ± 9.69	63.15 ± 10.05	63.84 ± 9.88	63.62 ± 9.53	<0.001
HDL-C, mg/dL, mean ± SD		42.34 ± 9.84	41.30 ± 10.79	41.46 ± 10.61	41.70 ± 10.94	42.34 ± 11.44	<0.001
LDL-C, mg/dL, median (Q1, Q3)		120 (97, 143)	122 (96, 148)	122 (96, 150)	121 (94, 151)	121 (94, 149)	0.053
Triglycerides, mg/dL, median (Q1, Q3)		124 (95, 167)	114 (87, 158)	117.00 (86, 160)	123 (89, 167)	129 (94, 178)	<0.001
Smoking or tobacco, n (%)		1127 (42.62%)	869 (34.50%)	794 (27.80%)	702 (26.51%)	610 (22.34%)	<0.001
Hypertension, n (%)		1038 (39.26%)	1056 (41.92%)	1373 (48.07%)	1366 (51.59%)	1494 (54.71%)	<0.001
PAD, n (%)		18 (0.68%)	12 (0.48%)	41 (1.44%)	25 (0.94%)	44 (1.61%)	<0.001
Prior TIA or stroke, n (%)		69 (2.61%)	59 (2.34%)	66 (2.31%)	65 (2.45%)	82 (3.00%)	0.480
Diabetes, n (%)		583 (22.05%)	614 (24.37%)	1207 (42.26%)	1770 (66.84%)	2381 (87.18%)	<0.001
NSTEMI, n (%)		1036 (39.18%)	907 (36.01%)	990 (34.66%)	989 (37.35%)	1005 (36.80%)	<0.001
STEMI, n (%)		1608 (60.82%)	1612 (63.99%)	1866 (65.34%)	1659 (62.65%)	1726 (63.20%)	<0.001
Killip class, n (%)							<0.001
	I	2353 (88.99%)	2266 (89.99%)	2450 (85.78%)	2184 (82.48%)	2224 (81.44%)	
	II	75 (2.84%)	109 (4.33%)	175 (6.13%)	173 (6.53%)	180 (6.59%)	
	III	134 (5.07%)	110 (4.37%)	180 (6.30%)	238 (8.99%)	267 (9.78%)	
	IV	82 (3.10%)	33 (1.31%)	51 (1.79%)	53 (2.00%)	60 (2.20%)	
LVEF category, n (%)							<0.001
	≤40%	231 (8.74%)	306 (12.15%)	418 (14.64%)	460 (17.37%)	524 (19.19%)	
	41% to 69%	1976 (74.74%)	1757 (69.75%)	1987 (69.57%)	1785 (67.41%)	1821 (66.68%)	
	≥70%	143 (5.41%)	177 (7.03%)	172 (6.02%)	139 (5.25%)	100 (3.66%)	
	Unknown or not assessed	294 (11.12%)	279 (11.08%)	279 (9.77%)	264 (9.97%)	286 (10.47%)	
LVEF, %, mean ± SD		53.40 ± 7.24	53.73 ± 7.75	53.43 ± 7.94	53.03 ± 7.75	52.33 ± 7.99	<0.001
Symptom onset to arrival (min), median (Q1, Q3)		256.5 (125, 960)	270 (120, 850)	255 (120, 830)	270 (120, 900)	270 (120, 885)	0.181
Antiplatelet, n (%)		2596 (98.26%)	2477 (98.33%)	2801 (98.11%)	2580 (97.69%)	2681 (98.31%)	0.392
Beta Blocker, n (%)		920 (36.74%)	918 (37.39%)	1070 (38.54%)	1009 (39.17%)	1011 (38.24%)	0.411
Cardiac arrest at admission, n (%)		13 (0.49%)	25 (0.99%)	43 (1.51%)	39 (1.47%)	40 (1.46%)	0.001
PCI, n (%)		968 (36.61%)	1366 (54.23%)	1604 (56.16%)	1459 (55.10%)	1429 (52.33%)	<0.001
MACEs, n (%)		86 (3.41%)	97 (3.67%)	116 (4.06%)	121 (4.57%)	173 (6.33%)	<0.001

PCI, percutaneous coronary intervention; PAD, peripheral artery disease; STEMI, ST-segment elevation myocardial infarction; NSTEMI, non-ST-segment elevation myocardial infarction; LVEF, left ventricular ejection fraction; MACEs, major adverse cardiovascular events; SBP, systolic blood pressure; HDL-C, high density lipoprotein cholesterol; LDL-C, low density lipoprotein cholesterol; TIA, transient ischemic attack; N, number of patients.

**Supplementary Table 1** presented baseline characteristics and crude outcomes according to MI type (NSTEMI vs. STEMI). There were significant inter-group differences in all variables except HDL-C. Patients with STEMI had a higher risk of 30-day MACEs in the fully adjusted model [OR (95% CI): 1.41 (1.11, 1.80), *p* = 0.005] (**Supplementary Table 2**).

### 3.2 Association between Glucose Level at Admission and 30-Day MACEs

As shown in Table 2, comparing with the reference (Q2, 5.4–6.3), participants with the highest quintile of glucose level were associated with increased risk of 30-day MACEs in the fully adjusted logistic regression model 3 [OR (95% CI): 1.82 (1.33, 2.50), *p* < 0.001]. Participants with a glucose reading ≥7.9 to ≤10.6 (Q4) had an increased risk of 30-day MACEs in unadjusted [OR (95% CI): 1.35 (1.02, 1.80), *p* = 0.035] and minimally adjusted [OR (95% CI): 1.35 (1.01, 1.80), *p* = 0.040] models, while the risk was not significant in the fully adjusted model [OR (95% CI): 1.22 (0.89, 1.69), *p* = 0.219]. Participants with glucose ≥6.4 to ≤7.8 (Q3) or ≤5.3 (Q1) had a higher but nonsignificant risk of 30-day MACEs, as compared with the reference Q2.

**Table 2.** **Relationship between glucose quintiles and 30-day MACEs in all participants**.

Glucose Quintiles	30-day MACEs		
	OR (95% CI), *p*-value		
	Model 1	Model 2	Model 3
All participants			
Q1	1.08 (0.80, 1.45) *p* = 0.621	1.06 (0.79, 1.44) *p* = 0.684	1.03 (0.75, 1.43) *p* = 0.835
Q2	reference	reference	reference
Q3	1.20 (0.90, 1.59) *p* = 0.213	1.17 (0.87, 1.56) *p* = 0.295	1.11 (0.81, 1.52) *p* = 0.519
Q4	1.35 (1.02, 1.80) *p* = 0.035	1.35 (1.01, 1.80) *p* = 0.040	1.22 (0.89, 1.69) *p* = 0.219
Q5	1.91 (1.47, 2.49) *p * < 0.001	1.94 (1.49, 2.54) *p * < 0.001	1.82 (1.33, 2.50) *p * < 0.001

Model 1: adjusted for none. Model 2: adjusted for intervention, age, sex and SBP. Model 3: adjusted for intervention, age, sex, SBP, weight, heart rate, HDL-C, LDL-C, TG, smoking or tobacco, diabetes, hypertension, PAD, MI Type, Symptom onset to arrival, Prior TIA or stroke, cardiac arrest at admission, LVEF category, PCI, antiplatelet, beta blocker. MACEs, major adverse cardiovascular events; SBP, systolic blood pressure; HDL-C, high density lipoprotein cholesterol; LDL-C, low density lipoprotein cholesterol; TG, triglyceride; PAD, peripheral artery disease; MI, myocardial infarction; TIA, transient ischemic attack; LVEF, left ventricular ejection fraction; PCI, percutaneous coronary intervention; OR, odds ratio; CI, confidence interval.

**Fig. 1.**
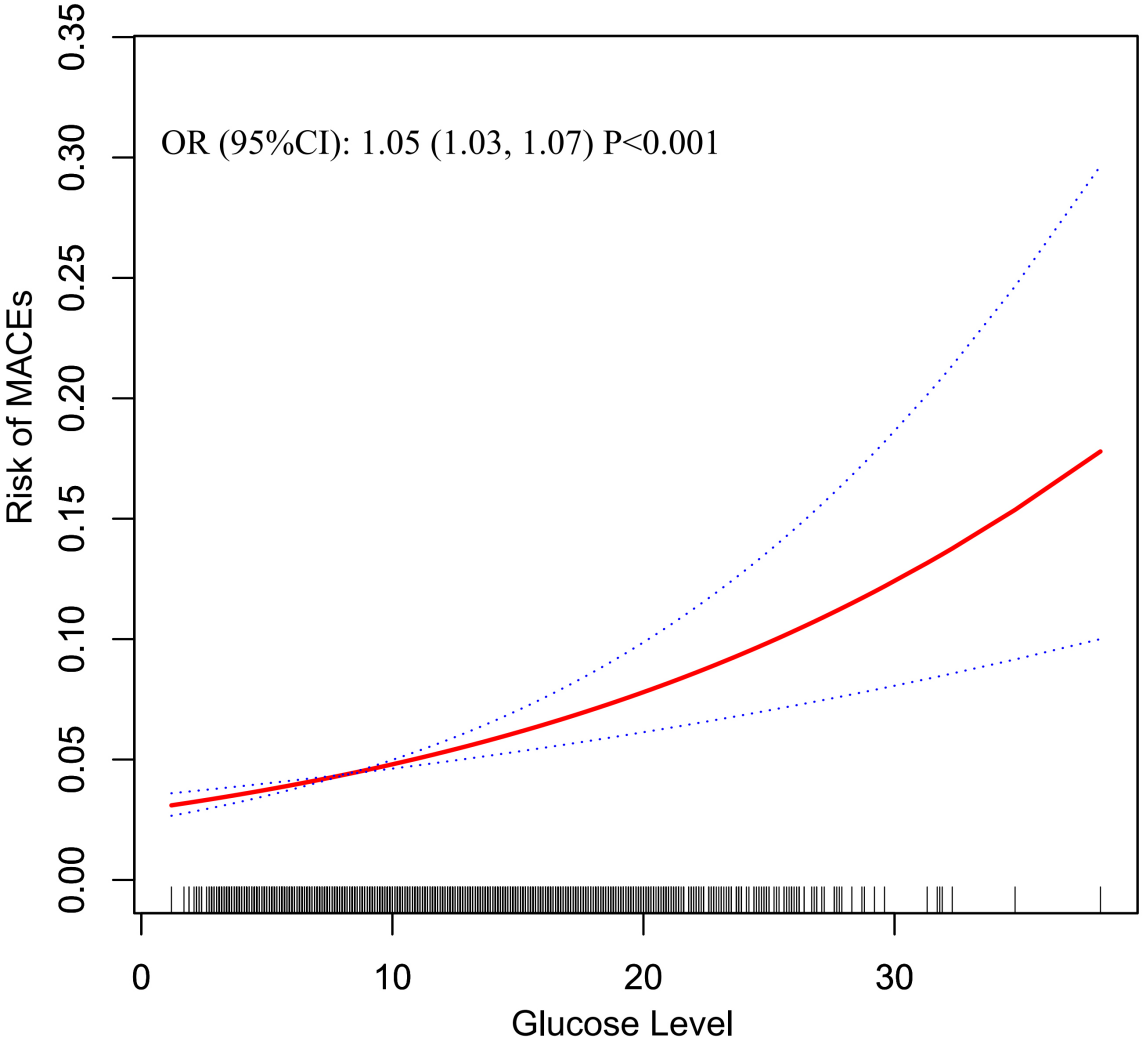
**Estimated risk of MACEs in different blood glucose levels for total participants**. The solid red line was the estimated risk, and the dashed lines above and below were the upper and lower limits of 95% CI, respectively. All covariates in model 3 were adjusted. MACEs, major adverse cardiovascular events; OR, odds ratio; CI, confidence interval.

**Fig. 2.**
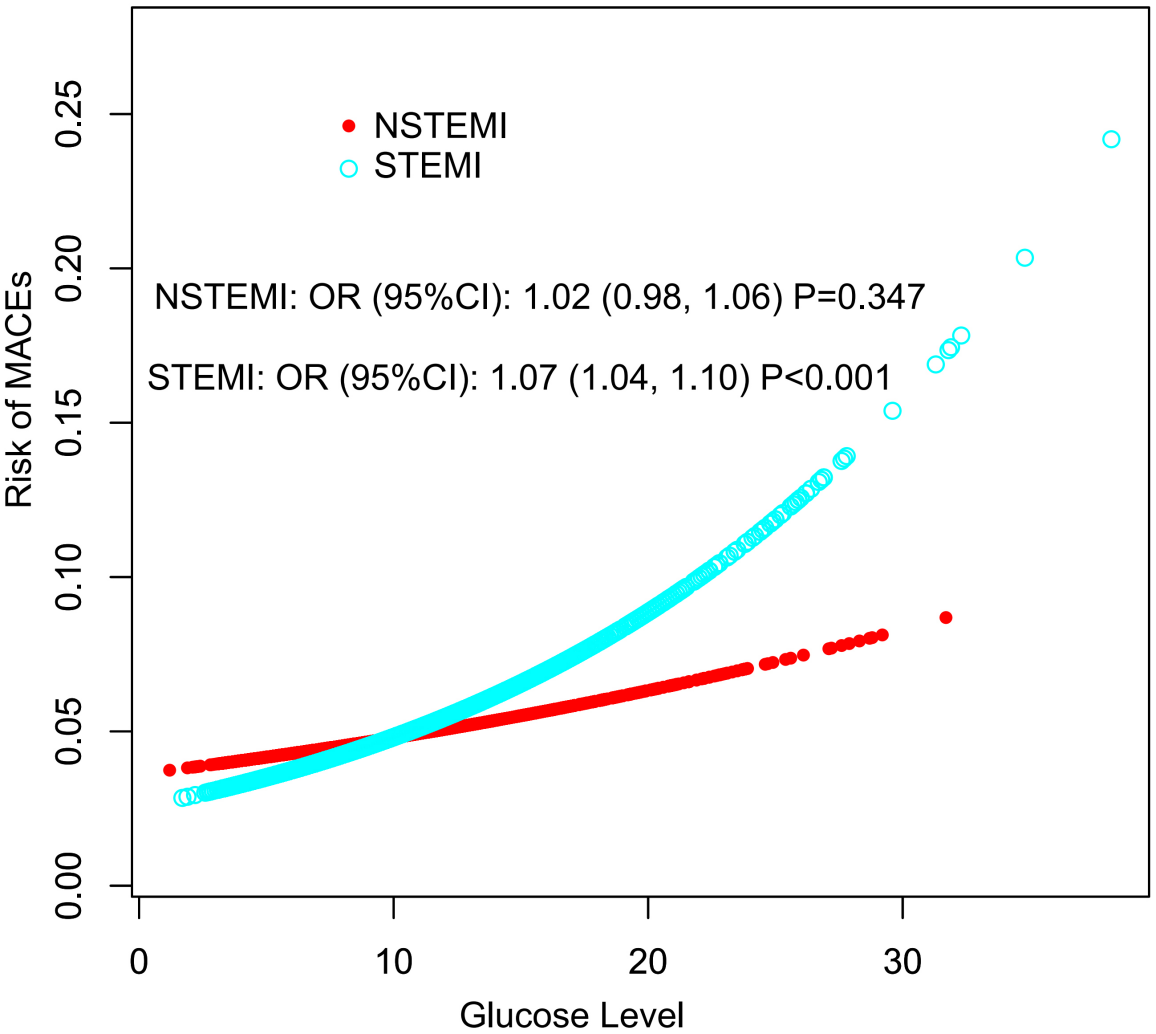
**Estimated risk of MACEs in different blood glucose levels stratified by MI type**. All covariates in model 3 were adjusted. MACEs, major adverse cardiovascular events; STEMI, ST-segment elevation myocardial infarction; NSTEMI, non-ST-segment elevation myocardial infarction; MI, myocardial infarction; OR, odds ratio; CI, confidence interval.

GAM was used to visualize the dose-response relationship between glucose level on admission and the risk of 30-day MACEs. As shown in Fig. 1, the risk of 30-day MACEs had a linear trend of increase with the increase of glucose. For each 1 mmol/L increase in blood glucose, the risk of 30-day MACES increased by 5% [OR (95% CI): 1.05 (1.03, 1.07)]. The parameters of other covariates in the Generalized additive model are shown in **Supplementary Table 3**. This trend was more significant in STEMI patients [OR (95% CI): 1.07 (1.04, 1.10)], with the risk of 30-day MACEs increasing with blood glucose levels more significant than in NSTEMI patients [OR (95% CI): 1.02 (0.98, 1.06)] (Fig. 2). The dose-response relationship between glucose level and the risk of 30-day MACEs was nearly flat trend (Fig. 2).

### 3.3 Subgroup Analyses and Interaction Test

The relationship between glucose level on admission and the risk of 30-day MACEs was still robust across the following subgroups (Table 3): age group (<65 vs. ≥65; *p* for interaction = 0.221), sex (female vs. male; *p* for interaction = 0.894), hypertension (no vs. yes; *p* for interaction = 0.421), diabetes (no vs. yes; *p* for interaction = 0.109).

**Table 3.** **Subgroup analysis of associations between 30-day MACEs and glucose quintiles among all participants**.

Subgroup		Glucose Quintiles					*p* for interaction
		Q1	Q2	Q3	Q4	Q5	
		OR (95% CI), *p*-value					
Age group							0.221
	<65	0.82 (0.48, 1.40) *p* = 0.465	reference	1.06 (0.64, 1.76) *p* = 0.825	1.48 (0.90, 2.46) *p* = 0.125	2.29 (1.39, 3.77) *p* = 0.001	
	≥65	1.21 (0.80, 1.83) *p* = 0.361	reference	1.19 (0.79, 1.78) *p* = 0.406	1.14 (0.75, 1.73) *p* = 0.543	1.70 (1.12, 2.57) *p* = 0.012	
Sex							0.894
	Female	1.08 (0.63, 1.85) *p* = 0.790	reference	1.25 (0.75, 2.08) *p* = 0.388	1.34 (0.80, 2.26) *p* = 0.268	1.76 (1.05, 2.96) *p* = 0.032	
	Male	1.06 (0.70, 1.59) *p* = 0.790	reference	1.05 (0.70, 1.59) *p* = 0.802	1.20 (0.80, 1.82) *p* = 0.380	1.96 (1.31, 2.94) *p* = 0.001	
Hypertension							0.421
	No	1.13 (0.70, 1.85) *p* = 0.614	reference	1.51 (0.95, 2.42) *p* = 0.083	1.58 (0.98, 2.55) *p* = 0.059	2.16 (1.33, 3.51) *p* = 0.002	
	Yes	0.98 (0.63, 1.52) *p* = 0.924	reference	0.89 (0.58, 1.37) *p* = 0.599	1.00 (0.64, 1.55) *p* = 0.999	1.60 (1.05, 2.44) *p* = 0.030	
Diabetes							0.109
	No	1.06 (0.71, 1.58) *p* = 0.791	reference	1.44 (0.97, 2.13) *p* = 0.072	1.53 (0.98, 2.38) *p* = 0.062	2.62 (1.57, 4.39) *p * < 0.001	
	Yes	1.03 (0.75, 1.43) *p* = 0.839	reference	1.13 (0.82, 1.54) *p* = 0.463	1.24 (0.90, 1.71) *p* = 0.190	1.86 (1.35, 2.55) *p * < 0.001	

All covariates in model 3 were adjusted except stratification itself. MACEs, major adverse cardiovascular events; OR, odds ratio; CI, confidence interval.

But, there was a significant interaction between glucose level and MI type in fully adjusted model 3 (NSTEMI vs. STEMI; *p* for interaction = 0.036). For example (Table 4), in STEMI patients, the highest quintile of glucose level (Q5) was significantly associated with an increased risk of 30-day MACEs after full adjustment [OR (95% CI): 2.23 (1.48, 3.35), *p* < 0.001]. But, this association was not significant in NSTEMI patients [OR (95% CI): 1.40 (0.84, 2.34), *p* = 0.191].

**Table 4.** **Relationship between glucose quintiles and 30-day MACEs in patients with STEMI and NSTEMI**.

Glucose Quintiles	30-day MACEs		
	OR (95% CI), *p*-value		
	Model 1	Model 2	Model 3
Patients with NSTEMI			
Q1	1.31 (0.83, 2.09) *p* = 0.251	1.31 (0.82, 2.09) *p* = 0.258	1.21 (0.73, 2.02) *p* = 0.452
Q2	reference	reference	reference
Q3	1.44 (0.91, 2.28) *p* = 0.121	1.34 (0.84, 2.14) *p* = 0.212	1.22 (0.73, 2.03) *p* = 0.442
Q4	1.04 (0.63, 1.70) *p* = 0.886	1.01 (0.62, 1.67) *p* = 0.955	0.85 (0.50, 1.47) *p* = 0.573
Q5	1.92 (1.24, 2.98) *p* = 0.004	1.83 (1.17, 2.85) *p* = 0.008	1.40 (0.84, 2.34) *p* = 0.191
Patients with STEMI			
Q1	0.93 (0.63, 1.37) *p* = 0.703	0.91 (0.61, 1.35) *p* = 0.634	0.89 (0.58, 1.36) *p* = 0.584
Q2	reference	reference	reference
Q3	1.07 (0.75, 1.54) *p* = 0.712	1.06 (0.74, 1.54) *p* = 0.746	1.04 (0.69, 1.56) *p* = 0.848
Q4	1.55 (1.10, 2.19) *p* = 0.013	1.56 (1.09, 2.21) *p* = 0.014	1.47 (0.98, 2.20) *p* = 0.061
Q5	1.91 (1.37, 2.66) *p * < 0.001	2.02 (1.44, 2.84) *p * < 0.001	2.23 (1.48, 3.35) *p * < 0.001
*p* for interaction	0.064	0.054	0.036

Model 1: adjusted for none. Model 2: adjusted for intervention, age, sex and SBP. Model 3: adjusted for intervention, age, sex, SBP, weight, heart rate, HDL-C, LDL-C, TG, smoking or tobacco, diabetes, hypertension, PAD, Symptom onset to arrival, Prior TIA or stroke, cardiac arrest at admission, LVEF category, PCI, antiplatelet, beta blocker. MACEs, major adverse cardiovascular events; STEMI, ST-segment elevation myocardial infarction; NSTEMI, non-ST-segment elevation myocardial infarction; SBP, systolic blood pressure; HDL-C, high density lipoprotein cholesterol; LDL-C, low density lipoprotein cholesterol; TG, triglyceride; PAD, peripheral artery disease; TIA, transient ischemic attack; LVEF, left ventricular ejection fraction; PCI, percutaneous coronary intervention; OR, odds ratio; CI, confidence interval.

## 4. Discussion

In this study, we found that AMI patients with elevated admission glucose have higher risks of 30-day MACEs, compared with patients who had normal levels of glucose on admission. There was a positive linear association between glucose levels on admission and the risk of 30-day MACEs in AMI patients. This trend was more significant in STEMI patients.

Elevated admission glucose levels were frequently reported to be an important factor of poor prognosis for patients with AMI, including increased risk of heart failure [17], hospital mortality [11], and left ventricular dysfunction [18]. A single-center prospective study showed that elevated blood glucose concentrations on admission were an independent prognostic factor for all-cause mortality in AMI patients [19]. The Cooperative Cardiovascular Project analyzed data on 141,680 patients aged 65 years or older with AMI and reported a positive linear association between the admission blood glucose level and the 30-day and 1-year mortality rates [20]. With the cut-off value of 110 mg/dL (6.0 mmol/L), this study reported a higher risk of 30-day and 1-year mortality for patients with higher admission glucose levels [20]. A prospective study on AMI patients found that compared to those with an admission glucose level lower than 140 mg/dL (7.8 mmol/L), patients whose admission glucose levels higher than 157 mg/dL (8.7 mmol/L) have a significantly higher risk of 30-day mortality [11]. Among ACS patients undergoing primary PCI, patients with admission glucose level ≥11.1 mmol/L, but not between 6.0 and 11.1 mmol/L had a higher risk of MACEs at 30 days [adjusted HR (95% CI): 5.21 (2.47, 10.98), *p* < 0.001], as compared to those <6.0 mmol/L [21]. Consistent with previous studies, in this study, we found that among AMI patients, the risk of 30-day MACEs was linearly increased as the admission blood glucose level increased. Compared to those with admission glucose levels of 5.4 mmol/L to 6.3 mmol/L, patients with admission glucose levels higher than 10.7 mmol/L have a significantly higher risk of 30-day MACEs.

Several hypotheses have been suggested to explain the relationship between elevated admission glucose levels and a higher risk of adverse cardiovascular outcomes in AMI patients. Elevated blood glucose levels might reflect a surge in stress hormones, such as catecholamines and cortisol, which produce an insulin-resistant state. It reduced glucose uptake by ischemic myocardium, increased circulating free fatty acids, and inhibited glucose oxidation, leading to increased membrane damage, arrhythmias, and reduced contractility [22, 23, 24, 25]. In addition, acute elevated blood glucose has been reported to have an association with increased thrombin formation, platelet activation, and fibrin clot resistance to lysis, which might increase the risk of thrombotic complications among AMI patients [26, 27]. Finally, previous clinical studies had reported that acutely elevated glucose was associated with left ventricular dysfunction, larger myocardial infarction size and higher risk of cardiogenic shock [18, 21, 28], which may directly explain the association between elevated glucose level and the increased risk of MACEs among AMI patients.

In addition, our analyses first reported a significant interaction between glucose level on admission and myocardial infarction type on the risk of 30-day MACEs (*p* for interaction = 0.036). In STEMI patients, the highest quintile of glucose level (Q5) was significantly associated with an increased risk of 30-day MACEs after full adjustment [OR (95% CI): 2.23 (1.48, 3.35), *p* < 0.001]. But, this association was not significant in NSTEMI patients [OR (95% CI): 1.40 (0.84, 2.34), *p* = 0.191]. Among patients with NSTEMI, the nonsignificant association between admission glucose level and the risk of 30-day MACEs might be explained by the following reasons. NSTEMI patients were older and more likely to have diabetes. Therefore, the elevated admission glucose in NSTEMI might not be the stress-induced increase in glucose following acute myocardial infarction, but rather the result of poor chronic glucose control [21, 29]. The degree of oxidative stress was closely related to acute rather than chronic fluctuations in blood glucose [30]. Besides, previous studies have reported that NSTEMI had a lower risk of in-hospital cardiovascular death and mortality at 2 months [31, 32]. In line with those findings, STEMI in this analysis had a higher risk of 30-day MACEs after adjusting for potential covariates [OR (95% CI): 1.41 (1.11, 1.80), *p* = 0.005], as compared to NSTEMI. However, NSTEMI patients were more likely to have a higher risk profile [32, 33, 34, 35]. Moreover, these risk factors can also predict CAD burden. Konrad Stepien *et al*. [36] interpreted the relationship between CAD burden and risk factors in NSTEMI patients. And NSTEMI in this study underwent less PCI and had a longer time from symptom onset to arrival. Those abovementioned high-risk characteristics might attenuate the relationship between glucose level on admission and the risk of 30-day MACEs among patients with NSTEMI.

The findings from this study have several clinical applications. First, we expanded current knowledge in the relationship between glucose levels on admission and risk of 30-day MACEs in AMI patients. The prognostic value of elevated admission glucose levels might be of substantial benefit in risk stratification and management of AMI patients. Second, by demonstrating the difference of this association between STEMI patients and NSTEMI patients, our study suggested that when managing AMI patients with high glucose levels on admission, more attention should be paid to STEMI patients than NSTEMI patients. Finally, to our knowledge, this was the largest retrospective study of this subject on the Indian population using ACS-QUIK data.

### Limitations

A few limitations of our study need to be addressed. First, given the nature of the retrospective study, it was possible that there was some residual confounding factors that were not measured. In addition, our study was limited to glucose levels on admission. Since data on glucose levels during hospitalization and follow-up information was not available, we were not able to assess whether the glucose levels were persistent during hospitalization. Above all, the proportion of AMI patients receiving standardized coronary reperfusion and drug therapy was limited in this study, which may affect the evaluation of the relationship between admission blood glucose and Mace. Future studies are warranted into the appropriate management of patients with AMI with high glucose levels on admission to hospital.

## 5. Conclusions

In conclusion, our results supported that elevated admission glucose level was a significant independent predictor of 30-day MACEs for AMI patients, especially in patients with STEMI. In clinical settings, more attention should be paid to STEMI patients with high admission glucose levels to prevent the occurrence of MACEs.

## Data Availability

Data are available from the Biologic Specimen and Data Repository Information Coordinating Center (BioLINCC), available on the National Heart, Lung and Blood Institute website (https://biolincc.nhlbi.nih.gov/studies/acs_quik/).

## Supplementary Material

Supplementary material associated with this article can be found, in the online version, at https://doi.org/10.31083/j.rcm2502046.
