# Supplementary material for: Elevated Glucose on Admission Was an Independent Risk Factor for 30-Day Major Adverse Cardiovascular Events in Patients with STEMI but Not NSTEMI
Source: Rev Cardiovasc Med. 2024 Jan 29;25(2):46. doi: 10.31083/j.rcm2502046 (PMC11263140; doi:10.31083/j.rcm2502046)
Supplement: Supplementary file 1 [file 2153-8174-25-2-046-s1.pdf]

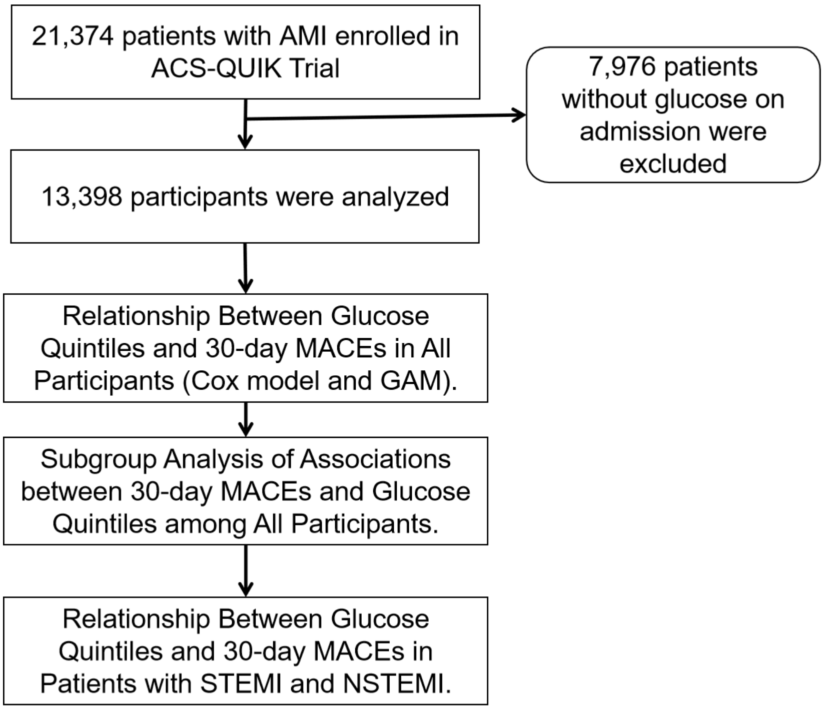

Supplementary Fig. 1. The flowchart of analysis in this study.

Supplementary Table 1. The diagnosis of multicollinearity in the covariates included in the fully adjusted logistic model.

Covariates	VIF
Intervention	1
Age	1.2
Male	1.3
Symptom onset to arrival	1
SBP	1.1
Weight	1.2
Smoking or tobacoo	1.2
Diabetes	1.4
Antiplatelet	1
Beta Blocker	1
PCI	1.1
Cardiac arrest at admission	1
Hypertension	1.2
prior TIA or stroke	1
PAD	1
LVEF category	1
MI type	1.3

Supplementary Table 2. Logistic regression results of each covariate and MACE.

Covariates	Term	OR	95%CI Low	95%CI Upp	P value
Intervention	factor (Intervention)	0.8159	0.7276	0.9149	0.0005
Age	Age	1.0424	1.0374	1.0475	<0.0001
Male	factor (Male)	0.5177	0.4594	0.5834	<0.0001
Symptom onset to arrival	Symptom onset to arrival	1	1	1.0001	0.0285
SBP	SBP	0.9844	0.9823	0.9865	<0.0001
Weight	Weight	0.9732	0.9673	0.9791	<0.0001
Smoking or tobacoo	factor (Smoking or tobacco)	0.7919	0.6957	0.9015	0.0004
Diabetes	factor (Diabetes)	1.4023	1.2505	1.5726	<0.0001
Antiplatelet	factor (Antiplatelet)	0.3644	0.2708	0.4903	<0.0001
Beta Blocker	factor (Beta Blocker)	0.5064	0.4433	0.5784	<0.0001
PCI	factor (PCI)	0.4205	0.3715	0.476	<0.0001
Cardiac arrest at admission	factor (Cardiac arrest at admission)	15.4705	12.2759	19.4965	<0.0001
Hypertension	factor (Hypertension)	1.2829	1.1439	1.4387	<0.0001
prior TIA or stroke	factor (prior TIA or stroke)	2.9795	2.2986	3.8621	<0.0001
MI type	factor (STEMI)	1.2858	1.1361	1.4553	0.0001

PAD	factor (PAD)	2.8644	1.9466	4.2151	<0.0001
LVEF category	factor (LVEF 41% to 69%)	0.2658	0.2316	0.3051	<0.0001
	factor (LVEF ≥70%)	0.2653	0.1919	0.3666	<0.0001

Supplementary Table 3. The parameters of other covariates in the Generalized additive model.

Covariates	P value	OR	95%CI lower limit	95%CI upper limit
Intervention	0.1063	0.8578	0.7121	1.0333
Age	<0.0001	1.0323	1.0238	1.0409
STEMI	0.0006	1.4377	1.169	1.7682
Male	<0.0001	0.5971	0.4794	0.7437
Symptom onset to arrival	0.0422	1.0001	1	1.0001
SBP	<0.0001	0.9936	0.9905	0.9967
Weight	0.3233	0.9949	0.9849	1.005
Smoking or tobacoo	0.1646	1.1808	0.9341	1.4926
Diabetes	0.8875	1.0153	0.8228	1.2529
Antiplatelet	0.8758	0.9592	0.5689	1.6172
Beta Blocker	0.0001	0.6636	0.5426	0.8115
PCI	<0.0001	0.4146	0.3352	0.5128
Cardiac arrest at admission	<0.0001	9.9922	6.6319	15.0551
Hypertension	0.4737	1.0752	0.8818	1.3111
prior TIA or stroke	<0.0001	2.2885	1.581	3.3125
PAD	0.0524	1.8641	0.9935	3.4977
factor(LVEF 41% to 69%)	<0.0001	0.4349	0.3506	0.5394
factor(LVEF ≥70%)	0.0001	0.35	0.2041	0.6003
